# Complex translocation disrupting *TCF4* and altering TCF4 isoform expression segregates as mild autosomal dominant intellectual disability

**DOI:** 10.1186/s13023-016-0439-6

**Published:** 2016-05-14

**Authors:** Valerie Maduro, Barbara N. Pusey, Praveen F. Cherukuri, Paul Atkins, Christèle du Souich, Rosemarie Rupps, Marjolaine Limbos, David R. Adams, Samarth S. Bhatt, Patrice Eydoux, Amanda E. Links, Anna Lehman, May C. Malicdan, Christopher E. Mason, Marie Morimoto, James C. Mullikin, Andrew Sear, Clara Van Karnebeek, Pawel Stankiewicz, William A. Gahl, Camilo Toro, Cornelius F. Boerkoel

**Affiliations:** NIH Undiagnosed Diseases Program, Common Fund, Office of the Director, National Institutes of Health, Bethesda, MD USA; Department of Medical Genetics, University of British Columbia, Children’s and Women’s Health Centre of BC, Vancouver, BC Canada; Child and Family Research Institute, University of British Columbia, Vancouver, BC Canada; Sunny Hill Health Centre for Children, Vancouver, BC Canada; Department of Molecular and Human Genetics, Baylor College of Medicine, Houston, TX USA; Department of Pathology and Laboratory Medicine, University of British Columbia, Vancouver, BC Canada; Department of Physiology and Biophysics, Weill Cornell Medical College, New York, NY USA; The HRH Prince Alwaleed Bin Talal Bin Abdulaziz Alsaud Institute for Computational Biomedicine, New York, NY USA; The Feil Family Brain and Mind Research Institute (BMRI), New York, NY USA; NIH Intramural Sequencing Center, National Human Genome Research Institute, National Institutes of Health, Bethesda, MD USA; Department of General Practice, Faculty of Medicine, University of British Columbia, Vancouver, BC Canada; NHGRI, National Institutes of Health, Bethesda, MD USA

**Keywords:** Intellectual disability, Promoter utilization, Pitt-Hopkins syndrome, TCF4, Gene expression, Translocation, Transcriptome, RNAseq

## Abstract

**Background:**

Mutations of *TCF4*, which encodes a basic helix-loop-helix transcription factor, cause Pitt-Hopkins syndrome (PTHS) via multiple genetic mechanisms. *TCF4* is a complex locus expressing multiple transcripts by alternative splicing and use of multiple promoters. To address the relationship between mutation of these transcripts and phenotype, we report a three-generation family segregating mild intellectual disability with a chromosomal translocation disrupting *TCF4*.

**Results:**

Using whole genome sequencing, we detected a complex unbalanced karyotype disrupting *TCF4* (46,XY,del(14)(q23.3q23.3)del(18)(q21.2q21.2)del(18)(q21.2q21.2)inv(18)(q21.2q21.2)t(14;18)(q23.3;q21.2)(14pter®14q23.3::18q21.2®18q21.2::18q21.1®18qter;18pter®18q21.2::14q23.3®14qter). Subsequent transcriptome sequencing, qRT-PCR and nCounter analyses revealed that cultured skin fibroblasts and peripheral blood had normal expression of genes along chromosomes 14 or 18 and no marked changes in expression of genes other than *TCF4*. Affected individuals had 12–33 fold higher mRNA levels of *TCF4* than did unaffected controls or individuals with PTHS. Although the derivative chromosome generated a *PLEKHG3*-*TCF4* fusion transcript, the increased levels of TCF4 mRNA arose from transcript variants originating distal to the translocation breakpoint, not from the fusion transcript.

**Conclusions:**

Although validation in additional patients is required, our findings suggest that the dysmorphic features and severe intellectual disability characteristic of PTHS are partially rescued by overexpression of those short *TCF4* transcripts encoding a nuclear localization signal, a transcription activation domain, and the basic helix-loop-helix domain.

**Electronic supplementary material:**

The online version of this article (doi:10.1186/s13023-016-0439-6) contains supplementary material, which is available to authorized users.

## Background

Intellectual disability (ID) is characterized as a significant deficit in intellectual functioning and in adaptive, conceptual, practical, and social skills [1], beginning before the age of 18 years. Depending on the ascertainment methodology and definition, the prevalence of ID in the general population is 1–3 % in industrialized countries [2–5].

Despite the prevalence and morbidity of ID, its physiologic bases remain poorly understood. Identified causes include environmental, epigenetic, and genetic factors [6, 7]. At a cellular level, these factors affect neuronal proliferation, migration, arborization, synaptogenesis, function, or viability [7–9].

Normal brain development involves the precise orchestration of several processes. Derailment of these processes by either a genetic or environmental insult causes cognitive and other neurodevelopmental disorders. Consistent with neurodevelopment being highly dependent on the choreographed expression of genes regulating neuronal development, an increasing number of cognitive disorders have been recently recognized to be attributable to mutations in regulators of gene expression [10–14].

Among the mutated chromatin regulators and transcription factors associated with ID is transcription factor 4 (TCF4). *TCF4* is transcribed from multiple promoters and alternative splice transcripts resulting in at least 18 different protein isoforms [15]. TCF4, via its interactions with other proteins, modulates an intricate combinatorial regulatory circuit during central nervous system (CNS) development [16]. Several splice variants show differential subcellular distribution [15]. *TCF4* encodes for class I basic helix-loop-helix (bHLH) proteins that function as transcriptional regulators when they heterodimerize with tissue-restricted class II bHLH proteins [17].

Class II bHLH transcription factors co-expressed or interacting with TCF4 during neurodevelopment include Math1, a proneural protein expressed in the differentiating neuroepithelium [18–20]; HASH1, a protein necessary for the formation of distinct neuronal circuits within the CNS, especially the telencephalon [21]; neuroD2, which plays important roles in neuronal differentiation and survival [22]; Id1, which is a homolog of proteins required for correct patterning in neurogenesis [23]; and Olig2, a regulator of ventral neuroectodermal progenitor cell fate [24–26].

Mono-allelic mutations or genomic deletions of *TCF4* cause Pitt-Hopkins syndrome (PTHS) [27–30]. PTHS has an estimated prevalence of 1 in 34,000 to 1 in 41,000 [31] and is characterized by severe ID, facial dysmorphism, episodes of hyperventilation, acquired microcephaly, seizures, happy disposition, and repetitive movements.

Analysis of the functional consequences of PTHS-associated *TCF4* mutations has found that not all deletions and truncations of TCF4 result in complete loss-of-function. Also, reading-frame elongating and missense mutations can cause a range of outcomes from subtle functional deficiencies to dominant-negative effects [30]. Consequently, PTHS-associated mutations variably impair the functions of TCF4 by diverse mechanisms and thereby contribute to the phenotypic variability. Herein, we further characterize the phenotypic variability and better define the molecular mechanisms underlying the ID associated with a balanced translocation interrupting *TCF4* and segregating with mild ID in three generations.

## Subjects

### Human subjects

The individuals or guardians of the individuals participating in this study gave informed consent approved by the Institutional Review Board (protocol 76-HG-0238) of the National Human Genome Research Institute. Two individuals with classic features of PTHS provided control blood and/or skin biopsy samples. They were a 14-year-old boy (UDP_10086; PTHS-1) with the mutation NM_001083962.1:c. [1650–1 G > A];[=], an established cause of PTHS and a splice acceptor mutation likely causing skipping of exon 18 and encoding p.Ser550Argfs*84 [32], and a 7-year-old girl (UDP_499; PTHS-2) with the mutation NM_001083962.1 (*TCF4*):c.[1726 C > T];[=] that encodes p.Arg576*.

### Clinical report

**Fig. 1:**
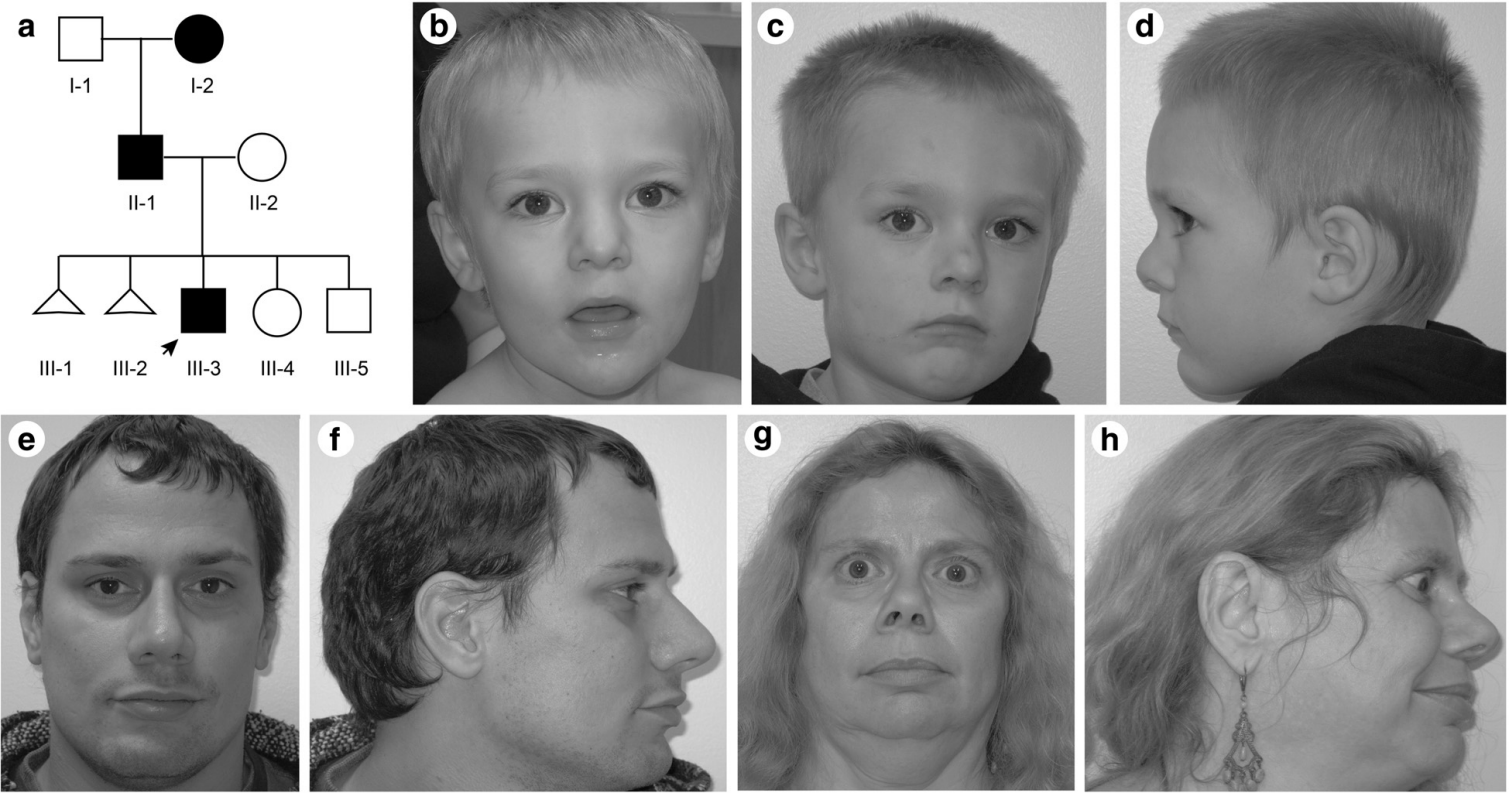
Clinical photographs of the proband (arrow, III-3), his affected father (II-1) and affected paternal grandmother (I-2). (**a**) Family pedigree. Affected individuals are shown by black symbols. (**b**, **c**) Frontal and profile head photographs of the proband at age 2.4 years. (**d**) Profile head photograph of the proband at age 3.4 years. (**e**, **f**) Frontal and profile head photographs of the proband’s father at age 30 years. (**g**, **h**) Frontal and profile head photographs of the proband’s paternal grandmother at age 53 years

The proband (UDP_4765; III-3, Fig. 1a) was born to non-consanguineous parents of mixed European descent and with a family history of miscarriages and intellectual disability. Exposures during the pregnancy included venlafaxine, a serotonin-norepinephrine reuptake inhibitor, and approximately 10 cigarettes per day. The proband was born at term following an uncomplicated pregnancy by spontaneous vaginal delivery. His birth weight, length and head circumference were 4.1 kg (92nd centile), 54.5 cm (99th centile), and 35 cm (66th centile), respectively. His Apgar scores were 9 at 1, 5 and 10 min. There were no neonatal complications or health problems during the first year of life.

At age 14 months, his parents noted delayed development. He scooted at about 12 months and walked without support at 18 ½ months. At the age of 2 years, he had 5 meaningful words and communicated predominantly by showing displeasure. At 27 months, his skills were at the level of a 15 to 18 month old; autism spectrum was ruled out. At 4 years 4 months, assessment with the Wechsler Preschool and Primary Scales of Intelligence – Third Edition (WPPSI-III) and Vineland Adaptive Behaviour Scale – Second Edition (Vineland-II), Survey Interview Form showed an uneven profile for verbal, nonverbal and language skills ranging from average to below average. His overall intellectual and adaptive functioning were below average for his age.

On physical examination at 28 months of age, he had diminished social interaction and had a height of 97.5 cm (98th centile), a weight of 15 kg (90th centile), and a head circumference of 50 cm (82nd centile). His dysmorphic features included plagio- and brachycephaly, prominent glabella, high anterior hairline, hypertelorism, upslanting palpebral fissures, bilateral epicanthal folds, bulbous nasal tip, prominent columella, large (6.0 cm, 97th centile) cupped ears with a simple helix, a high arched palate, a prominent chin, mildly hypoplastic zygomatic arch, and a pectus carinatum (Fig. 1b-d). He also had a left single palmar crease, prominent finger pads, 5th finger clinodactyly, bilateral hallux valgus and clinodactyly of toes 3 to 5. On neurologic exam he had normal strength and deep tendon reflexes, mildly decreased central tone and a wide based gait.

The proband’s father (UDP_4637; II-1, Fig. 1a) had a similar history of developmental delay and impaired speech development. He finished high school with assistance and worked in a fast food restaurant. Formal neurocognitive testing at age 31 years using Wechsler Adult Intelligence Scale, Fourth Edition (WAIS-IV) and Adult Self-Report (ASR) for Ages 18–59 revealed mild intellectual disability with nonverbal reasoning significantly lower than verbal reasoning. On physical exam, his height was 173 cm (31st centile), and his head circumference was 57 cm (56th centile). His dysmorphic features included mild plagiocephaly, a high forehead, high anterior hairline, upslanting palpebral fissures, simple ear helices, prominent chin, high arched palate, left single palmar crease, and prominent finger pads (Fig. 1e, f). His neurologic examination was normal.

The proband’s paternal grandmother (UDP_4638; I-2, Fig. 1a) had clinical depression and had undergone multiple surgeries for keratoconus. Her height measured 158.4 cm (23rd centile), and her head circumference was 53.5 cm (22nd centile). She had a high forehead, bulbous nasal tip, and mild proptosis (Fig. 1g, h). At age 53 years, her neurologic examination was unremarkable. Formal testing by WAIS-IV, ASR and Wechsler Memory Scale – Fourth Edition (WMS-IV) revealed a mild intellectual disability as well as verbal and visual memory impairments.

#### Results of additional investigations

Normal laboratory investigations for the proband included a complete blood count and blood electrolytes, lipid profile, liver and kidney function tests, and blood levels for ammonia, lactate, thyroid stimulating hormone and gonadotropic steroids. He also had unremarkable plasma amino acid and urine organic acid profiles and a normal skeletal survey and bone age. He tested negative for an *FMR1* repeat expansion. Chromosome analysis revealed an apparently balanced translocation, 46,XY, t(14;18)(q22;q21) and chromosomal microarray analysis (GenomeDXv2.0) found no clinically significant copy number variants. The proband’s father and paternal grandmother had the same chromosome translocation. A brain MRI performed on the proband’s father showed no structural or myelination abnormalities.

## Methods

Characterization of the cytogenetically identified translocation and delineation of the potential mechanism of disease was conducted by a series of molecular analyses that included whole genome and transcriptome sequencing followed by validation studies (Additional file 1: Figure S1).

### Nucleic acid extraction

Genomic DNA was extracted from peripheral whole blood using the Gentra Puregene Blood kit (Qiagen, Valencia, CA) per the manufacturer’s protocol. Total RNA was extracted from cultured skin fibroblasts using the RNeasy Mini Kit (Qiagen, Valencia, CA) per the manufacturer’s protocol. Total RNA from patient and control peripheral whole blood samples was purified using the QuickGene 810 automated extraction machine (Autogen, Holliston, MA) with an on column DNase digestion. The quality and quantity of RNA was verified using an Agilent 2100 Bioanalyzer (Agilent Technologies, Santa Clara, CA) and NanoDrop 8000 (Thermo Scientific, Waltham, MA).

### SNP Chip analysis

The Illumina GenomeStudio^TM^ software (V2011.1, Illumina, San Diego, CA) was used to define the population frequency of the B allele (PFB) statistics for 662 samples from unrelated Undiagnosed Diseases Program (UDP) individuals. Samples were run on the Illumina Human OmniExpress-12v1_A chip and the resulting PFB file was filtered for mitochondrial and chromosomal 0 SNP data. Post-filtering, GenTrain score (clustering algorithm score), genotype, B Allele Frequency (BAF), and log R Ratio (LRR) for the proband were generated and exported. The proband input file was run against the filtered PFB file using PennCNV [33] with thresholds of 2, 5, or 10 SNPs to generate threshold specific copy number variant (CNV) calls.

All CNV calls were manually inspected and validated for accuracy. Each copy number (CN) call position was entered into the Illumina Genome Viewer (GenomeStudio^TM^) and inspected with BAF and LRR plots for the proband. Call authenticity was verified by comparing normalized intensity of the A and B allele Cartesian coordinates of the proband to rest of population in the dataset. Illumina GenomeStudio^TM^ Genotyping Module generated normalized intensity values.

### Whole-genome sequencing

Patient blood genomic DNA libraries were prepared and sequenced according to Illumina (Illumina., San Diego, CA) paired-end sequencing service protocols. Illumina’s service package consisted of short-insert (308 median fragment length) paired-end reads from one library with 100 bp read length. The library was barcoded and sequenced on 2 flow-cells (3 lanes) of Illumina HiSeq2000 platform and produced >89 billion high-quality bases (Additional file 2: Table S1). Preliminary bioinformatics alignment analysis of the whole-genome sequencing data was based on the Illumina pipeline (CASAVA 1.8). CASAVA performed multi-seed and gapped alignments on human reference sequence (NBCI Build 37; hg19). Sequences with more than two mismatches and duplicated sequences corresponding to PCR amplification bias were excluded (Additional file 3: Table S2). This left a total of 3,697,786 SNVs with a heterozygous : homozygous non-reference ratio of 1.5 (Additional file 1: Table S3).

### Detection of structural variations from whole-genome sequence data

Inter- and intra-chromosomal structural variations (SVs) from the Illumina ELAND alignments were detected with BreakDancer (version 1.1) and an in-house program, BREAKER (Cherukuri PF, et al. unpublished data); SVs were called with stringent criteria (−q 35 -r 2). BreakDancer calls were filtered to include only SV calls in which either plus or minus strand reads were at maximum 60 at both breakpoints and were supported by at least 12 plus or minus strand reads. The maximum cutoff was performed to discard regions with suspiciously high sequencing depth. BreakDancer calls with scores of 99 and higher were included in further analysis. These high confidence SV calls were filtered against (1) DGV high-throughput sequencing variants (UCSC track table), (2) Segmental Duplications (UCSC track table), and (3) HiSeq depth regions (top 5 % UCSC track table). In steps 1, 2 and 3, an SV call was filtered out if at least one of the breakpoints was located within a ±500 bp window of a repetitive genomic region (in case of a translocation, a 1001 bp window centered on the breakpoint). These SV candidate calls were visually inspected with IGV and validated. This methodology found 22 putative insertions, deletions and inversion candidates. Of these candidates, 5 were within genes (4 autosomal; 1 X-linked), and two interrupted a protein coding sequence: *MIER1* and *QPCT*. Eighty-four percent of the BreakDancer calls were manually assessed as false positives after the systematic filtering. Single short-reads mapped across the candidate inter-chromosomal translocation break-point: chr14:chr18.

### Tissue culture

Skin fibroblasts were obtained from skin biopsies. Both affected fibroblasts and unaffected control fibroblasts were grown in high-glucose DMEM medium with L-glutamine (Life Technologies, Carlsbad, CA) supplemented with 10 % fetal bovine serum and 1 % Antibiotic-Antimycotic (Life Technologies, Carlsbad, CA). Cultured fibroblasts were incubated in a humidity-controlled environment at 37 **°**C, with 95 % O_2_ and 5 % CO_2_. The medium was exchanged for fresh medium every 3 days, and the cells were used before passage 10.

### RNA-seq Method

Poly-A selected RNA-seq libraries were constructed from 1 μg mRNA using the Illumina TruSeq RNA Sample Prep Kits, version 2 (Illumina, San Diego, CA). The resulting cDNA was fragmented using a Covaris E210. Library amplification was performed using 8 cycles to minimize the risk of over-amplification. Unique barcode adapters were applied to each library. Libraries were quantitated by qPCR using the KAPA Library Quantification Kit (KAPA Biosystems) and pooled in an equimolar ratio. The pooled libraries were sequenced on a GA_ii_x. At least 40 million 101-base read pairs were generated for each individual library. Data were processed using RTA 1.12.4.2 and CASAVA 1.8.2.

### Transcriptome data processing and data analysis

Transcriptome fastq reads (phred33-scaled) were mapped onto the human genome assembly hg19 using Bowtie2 in TopHat2 (v.2.0.3) [34, 35]. Pre-computed human reference sequence (NBCI Build 37; hg19) Bowtie2 index files were used as the index files for read mapping. The UCSC known gene splice junction library (GTF file) was used for splice-read mapping; in addition, the fusion-search parameter switch was turned on to enable gene-fusion derived transcript discovery. Transcript assembly, abundance estimates and differential expression analyses were performed using Cufflinks2 (v2.2.1) and Cuffdiff2 (v2.2.1) [35, 36]. Differential gene expression comparisons were run without biological replicates; therefore biological sample gene variance could not be estimated. Differential expression was calculated as fold-changes in gene expression (measured as fragments per kilobase mapped (FPKM)). Pseudo-count of FPKM 1 was added to all FPKM values to minimize inflation of differential gene expression log-likelihood ratios (base 10). Local neighborhood gene-differential analysis was performed at chromosomal breakpoint junctions, using Pearson correlation coefficient to detect anti-correlated gene expression signature deviation from expectation.

### Analysis of gene expression on chromosomes 14 and 18

The Pearson correlation coefficient of gene expression on chromosomes 14 and 18 was calculated using all-possible pairs (*N*^*2*^) resulting from a window of 3 genes. The methodology is described in the Additional file 1.

### PCR amplification

Genomic DNA sequences of interest were amplified by polymerase chain reaction using the listed primers (Additional file 1: Table S4), genomic DNA and Qiagen HotStar Plus Taq polymerase under conditions: 95 °C x 5 min denaturation followed by 40 cycles of 95 °C x 30 s, 55 °C x 30 s, 72 °C x 30 s.

### Sanger sequencing

Residual primers and nucleotides were removed by incubation with ExoSAP-IT (USB, Cleveland, OH). The amplicons were sequenced by Macrogen (Rockville, MD) using BigDye terminator chemistry and compared to the human reference sequence (NCBI 37/hg19) using Sequencher (GeneCodes, Ann Arbor, MI).

### Reverse transcription polymerase chain reaction and quantitative real-time polymerase chain reaction

For cultured fibroblasts, complementary DNA (cDNA) synthesis was performed on 2 μg of total RNA using the OmniScript RT Synthesis kit (Qiagen, Valencia, CA) and Oligo dT_23_ Anchored Primers (Sigma, St. Louis, MO). The cDNA sequence was verified by PCR analysis using the listed primers (Additional file 1: Table S5), HotStar Plus Taq polymerase (Qiagen, Valencia, CA) and 100 ng of cDNA under conditions: 95 °C × 5 min denaturation followed by 40 cycles of 95 °C × 30 s, 60 °C × 30 s, 72 °C × 30 s.

For peripheral blood, cDNA synthesis was performed on 40 ng of total RNA using the SensiScript RT kit (Qiagen, Valencia, CA) and Oligo dT_23_ Anchored Primers (Sigma, St. Louis, MO). The cDNA sequence was verified by PCR analysis using the listed primers (Additional file 1: Table S5), HotStar Plus Taq polymerase (Qiagen, Valencia, CA) and 80 ng of cDNA under conditions: 95 °C × 5 min denaturation followed by 40 cycles of 95 °C × 30 s, 60 °C × 30 s, 72 °C × 30 s.

Quantitative real-time PCR was performed on 80 ng of cDNA, the listed primers (Additional file 1: Table S5) and the QuantiFast SYBR Green PCR Kit (Qiagen, Valencia, CA), and analyzed with the ABI 7500 Fast Real-Time PCR System (Life Technologies, Carlsbad, CA). Target amplification was normalized to that of *GAPDH* and shown as expression relative to control.

### Digital droplet PCR

Digital droplet PCR analysis was performed on 50 ng of cDNA derived from patient and control fibroblast RNA using TaqMan Genotyping Mastermix (Life Technologies, Carlsbad, CA) and TaqMan Gene Expression Assay for rs1261084 (Life Technologies, Carlsbad, CA) under conditions: 95 °C x 10 min denaturation followed by 40 cycles of 95 °C × 15 s, 60 °C × 60 s both with a ramp speed of 0.5 °C per second. The amplified products (4 million droplets per sample) were read on the RainDrop Digital PCR System (RainDance Technologies, Billerica, MA) and analyzed using the Raindrop Analyst software. Results were normalized to control fibroblasts.

### nCounter gene expression assay

The nCounter Gene expression assay was performed on 100 ng of total RNA derived from human blood peripheral leukocytes or cultured fibroblasts from the patient, PTHS controls, and unaffected controls (Clontech, Mountain View, CA). The RNA samples were hybridized at 65 °C for a minimum of 12 h to the Capture and Reporter probesets (nanoString Technologies, Seattle, WA) that were designed to include the listed *TCF4* transcript variants (Additional file 1: Table S6). These complexes were immobilized onto a cartridge and analyzed by the nCounter Digital Analyzer (nanoString Technologies, Seattle, WA). Geometric means were used to calculate the normalization factor and data were normalized to *GAPDH* expression. The results were analyzed, calculated relative to control gene expression in blood derived samples, and reported as the log_2_ ratio relative to control *TCF4* transcript levels.

## Results

### TCF4 is disrupted by a complex chromosomal translocation that segregates with ID in three generations

**Fig. 2:**
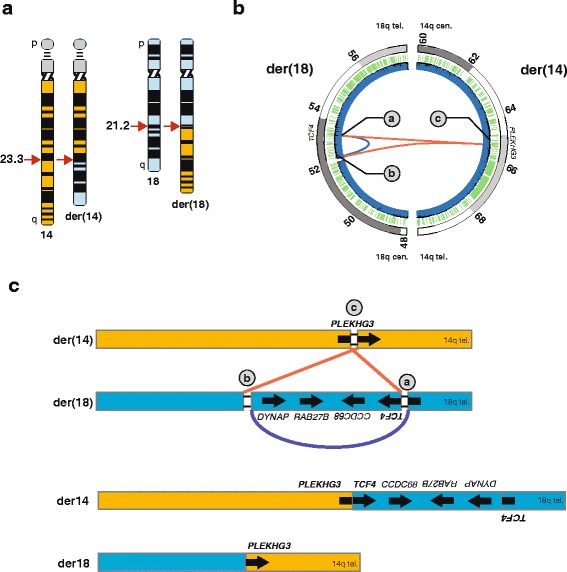
Delineation of a balanced translocation (t(14;18)) disrupting transcription factor 4 (*TCF4*) using whole genome sequencing of patient DNA. (**a**) Ideogram depicting the patient’s apparently balanced translocation t(14,18)(q23.2;q21.2) and normal karyotype (46, XY). The ideogram of chromosome 18 is shaded in *light blue* color. (**b**) Inter-chromosome (*red;* chr18-chr14) and intra-chromosome (*blue;* chr18-chr18) connections identified by whole-genome sequence analysis. Intra-chromosomal inversion (943,387 bases) on chr18 encompasses *RAB27B,* and *CCDC68* and interrupted *DYNAP* and *TCF4.* The inversion junctions are flanked by heterozygous deletions within *TCF4* (19,394 bases (**a**)) and include the promoter and first exon of *DYNAP* transcript NM_173629 (38,926 bases (**b**)). Inter-chromosome connection on chr14 disrupts *PLEKHG3* resulting in a 29 bp heterozygous deletion (**c**). *Blue* and *orange* arrows indicate genes on the positive and negative strand respectively. *Dark blue* and *green* wiggles indicate read depth via whole-genome sequencing, and segmental duplications (hg19 UCSC Human genome browser) respectively. (**c**) Schematic representation mechanism of the three ds-DNA breaks and genomic reorganization steps that led to the translocation event between chromosome 14 and 18. The three main steps were: (1) a 0.94 Mb inversion (blue arch, breakpoints **a** and **b**) on chromosome 18, followed by (2) ligation of the centromeric portion of chromosome 14 (red line, breakpoints **c** and **a**) with the telomeric q arm of chromosome 18 to yield der (14), and (3) ligation of the centromeric portion of chromosome 18 (red line, breakpoints **c** and **b**) ligation with the telomeric q arm of chromosome 14 to yield der (18). The schematic representation of chromosomes is not to scale. The der (14) chromosome harbors a gene fusion of *PLEKHG3* (5’ untranslated region) and *TCF4* (coding exons) as well as interrupted *TCF4* transcript variants. The der (18) chromosome harbors a disrupted copy of *PLEKHG3*; the coding potential of the gene remains intact although the promoter and first non-coding exon are removed by the translocation

To identify genes disrupted by the apparently balanced translocation between chromosomes 14 and 18, we generated a 308 bp-insert Illumina whole-genome sequencing library for whole genome sequencing. The 100 bp paired-end sequencing of whole blood DNA generated 1,094,407,124 individual reads with 452 million high-quality pairs. Analysis of aligned pairs identified a cluster of reads with ends mapping to chromosome 14 and 18. From this analysis, 30 read pairs with high-quality mapping localized to a single origin in the first intron of *PLEKHG3* (NM_015549.1) on chromosome 14 (chr14: 65,191,597-65,191,620) (Fig. 2a), and 29 bp (chr14: 65,191,595-65,191,623) were deleted at the breakpoint (Additional file 1: Figure S1).

On chromosome 18 (18q21.2), the cluster-signal split nearly in half (14 read-pairs, and 16 read-pairs) and mapped to two distinct locations 0.98 Mb apart (Fig. 2a). Further analysis of reads from this region of chromosome 18 suggested an inversion of 18q21.2. This inversion (0.98 Mb, chr18: 52,256,629- 53,200,017) encompassed *RAB27B, CCDC68,* and interrupted *TCF4* and *DYNAP* (Fig. 2b, Additional file 1: Figure S2). The centromeric breakpoint deleted 38.9 Kb (chr18: 52,217,704-52,256,628) including the promoter and first exon of *DYNAP* transcript NM_173629. The telomeric breakpoint deleted 19.4 Kb (chr18: 53,200,018-53,219,411) within *TCF4*; this did not delete any defined promoters or exons for transcripts of *TCF4*. We confirmed the inversion breakpoints (chr18: 53,200,017) by PCR amplification and Sanger sequencing (Fig. 2b, Additional file 1: Figure S2).

**Fig. 3:**
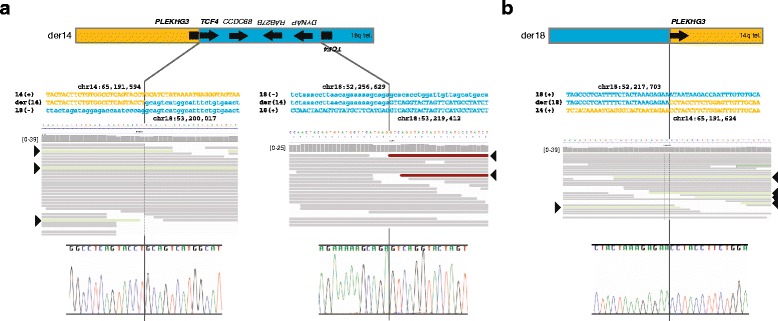
Characterization of the breakpoints giving rise to the derivative chromosomes 14 and 18 using massively parallel whole-genome sequencing and Sanger sequencing. (**a**) Characterization of derivative chromosome 14 and its breakpoints. The top panel shows a graphic of the derivative chromosome. The middle panel shows the sequence of chromosome 14 (orange type), chromosome 18 (blue type), the derivative chromosome, and the pileup of whole-genome sequencing reads at each junction. Forward sequence is shown as uppercase letters and reverse sequence as lowercase letters. The mate pairs spanning the translocation junction are shown in *light green* (arrowheads), and those spanning the inversion junction are shown in *red* (arrowheads). The lower panel shows the chromatogram for Sanger sequencing across the junction. (**b**) Characterization of derivative chromosome 18 and its breakpoints. The top panel shows a graphic of the derivative chromosome. The middle panel shows the sequence of chromosome 14 (orange font), chromosome 18 (blue font), the derivative chromosome, and the pileup of whole-genome sequencing reads at each junction. Forward sequence is shown as uppercase letters and reverse sequence as lowercase letters. The mate pairs spanning the translocation junction are shown in *light green* (arrowheads). The lower panel shows the chromatogram for Sanger sequencing across the junction

On one derivative chromosome, the portion of chromosome 14 centromeric to the *PLEKHG3* intron 1 breakpoint (chr14: 65,191,597) was joined to the breakpoint of the inverted terminal portion of *TCF4* (chr18: 53,200,017) and the telomeric portion of 18q (Fig. 3a, Additional file 1: Figure S2)*.* On the second derivative chromosome, the portion of chromosome 18 centromeric to the breakpoint within *DYNAP* (chr18: 52,217,703) was joined to the portion of 14q telomeric to the *PLEKHG3* intron 1 breakpoint (chr14: 65,191,620) (Fig. 3b, Additional file 1: Figure S2). These findings give a revised karyotype of 46,XY,del(14)(q23.3q23.3)del(18)(q21.2q21.2)del(18)(q21.2q21.2)inv(18)(q21.2q21.2)t(14;18)(q23.3;q21.2)(14pter®14q23.3::18q21.2®18q21.2::18q21.1®18qter;18pter®18q21.2::14q23.3®14qter.

### The translocation does not disrupt global gene-expression patterns on the derivative chromosomes (der14 and der18)

Given the observation that patients in our study did not share the distinctive features of Pitt Hopkins syndrome (Table 1), the syndromic form of ID associated with heterozygous *TCF4* mutations, and the potential for translocated chromosomal segments to have altered gene expression [37], we used quantitative RNA sequencing to test for gene expression changes on chromosomes 14 and 18 (see Methods). Using RNA extracted from cultured skin fibroblasts of individual II-1 and matched controls, we generated libraries and performed 101 bp paired-end transcriptome sequencing. This generated 114,477,006 (II-1) and 112,237,295 (control) high-quality reads for processing and evaluation using standard bioinformatics methodologies [34, 35]. Since chromosomal rearrangements can disrupt the spatial connection between a gene and its regulatory elements [38], we asked whether there were detectable patterns of gene-misregulation on the derivative chromosomes by computing the cross-correlation of all genes (Pearson correlation coefficient) along chromosomes 14 (755 genes) and 18 (324 genes). We generated the Pearson correlation coefficient matrix (*M*_*i,j*_) for all pairs of genes and evaluated the topological overlap along the diagonal for signatures of anti-correlation along the entire length of chromosomes 18 and 14 (Additional file 1: Figure S3A, B). Data from experiments did not reject the null hypothesis, suggesting that any observed alterations in gene expression were random. Nonetheless, to characterize further the local expression ordering, we performed window-modularity gene-expression analysis by comparing expression between patient and control fibroblasts [39, 40]. These analyses also did not reveal statistically significant differences in gene-expression patterns. We concluded, therefore, that gene expression changes across large regions of chromosomes 14 and 18 were either unlikely to be the cause of this patient’s phenotype or were undetectable in cultured fibroblasts.

**Table 1:** Comparison of our patients’ features to individuals reported with *TCF4* mutations

Feature	Summary of reported patients [27–29, 31, 43–45, 49–61]			Our patients	
	No. reported with feature	Total reported (*n* = 121)	%	No. with feature	Total (*n* = 3)
*Pitt Hopkins facial gestalt*	109	114	96	0	3
Deep set eyes	85	103	83		
Protrusion of mid/lower face	96	105	91		
Marked nasal root	90	105	86		
Broad/beaked bridge	99	106	93		
Flared nostrils	90	106	85		
Large mouth	101	109	93		
Tented upper lip	102	107	95		
Everted lower lip	94	107	88		
*Hands*					
Long fingers	26	46	57	1	3
Single palmar crease	52	88	59	3	3
Prominent finger pads	31	69	45	2	3
Additional palmar creases	5	32	16	0	3
Thumb ankylosis	10	68	15	0	3
*Feet*					
Pes planus	15	25	60	1	1
Pes cavus	4	22	18	0	1
Overriding toes	15	38	39	0	1
Talipes equivarus2	6	24	25	0	3
*Genitalia*					
Abnormal	21	57	37	0	1
Cryptorchidism	14	42	33	0	1
*Spine*					
Scoliosis	15	79	19	0	3
*Ophthalmological findings*	73	104	70		
Strabismus	65	113	58	0	3
Myopia	43	86	50	2	3
*Gastrointestinal findings*	17	26	65	0	3
Constipation	66	101	65		
GER	8	44	18		
Hirschsprung disease	1	74	1		
*Growth findings*				0	3
Height < 2 SD	19	80	24		
Weight < 2 SD	9	58	16		
OFC < − 2 SD	17	65	26		
OFC on −2 SD	13	49	27		
Microcephaly	56	113	50		
*Developmental findings*					
Severe ID or DD	117	119	98	0	3
Hypotonia	69	80	86		
Delayed walking	81	83	98	0	1
Walking achieved	53	65	82	3	3
Ataxic gait	44	68	65	0	3
Absent language or <5 words	105	108	97	0	3
*Movement anomalies*	81	106	76	0	3
Arm flapping	28	47	60		
Hand biting/nibbling	17	38	45		
Repetitive finger movements	12	25	48		
Repetitive wrist movements	10	21	48		
Hand wringing	11	24	46		
Head stereotypies	11	26	42		
Median line stereotypies	14	35	40		
*Behavioral findings*				0	3
Smiling/happy	86	99	87		
Harm to self	21	59	36		
Harm to others	12	61	20		
Sleep disturbances	15	63	24		
Anxiety/agitation	29	49	59		
Unmotivated laughing	19	30	63		
*Breathing anomalies*	72	116	62	0	3
Hyperventilation	54	99	55		
Apnoea	35	65	54		
Cyanosis	11	36	31		
Loss of consciousness	5	34	15		
Chronic hypoxia	3	31	10		
Finger clubbing	3	33	9		
*Seizures (history)*	44	113	39	0	3
*Abnormal head MRI*	59	95	62	0	1
Hypoplasia or agenesis of cc	36	83	43		
Ventriculomegaly	24	71	34		
Abnormal myelination or reduced white matter	5	29	17		
Cortical atrophy	6	37	16		
Minor posterior fossa anomalies	6	22	27		
Dentate nuclei hyperintensity	4	25	16		
Small hippocampi	11	41	27		
Temporal lobe hyperintensity	13	51	25		
Frontal lobe hypoplasia	3	30	10		
Large caudate nuclei	4	45	9		
*Normal birth parameters*	36	41	88	1	1

**Fig. 4:**
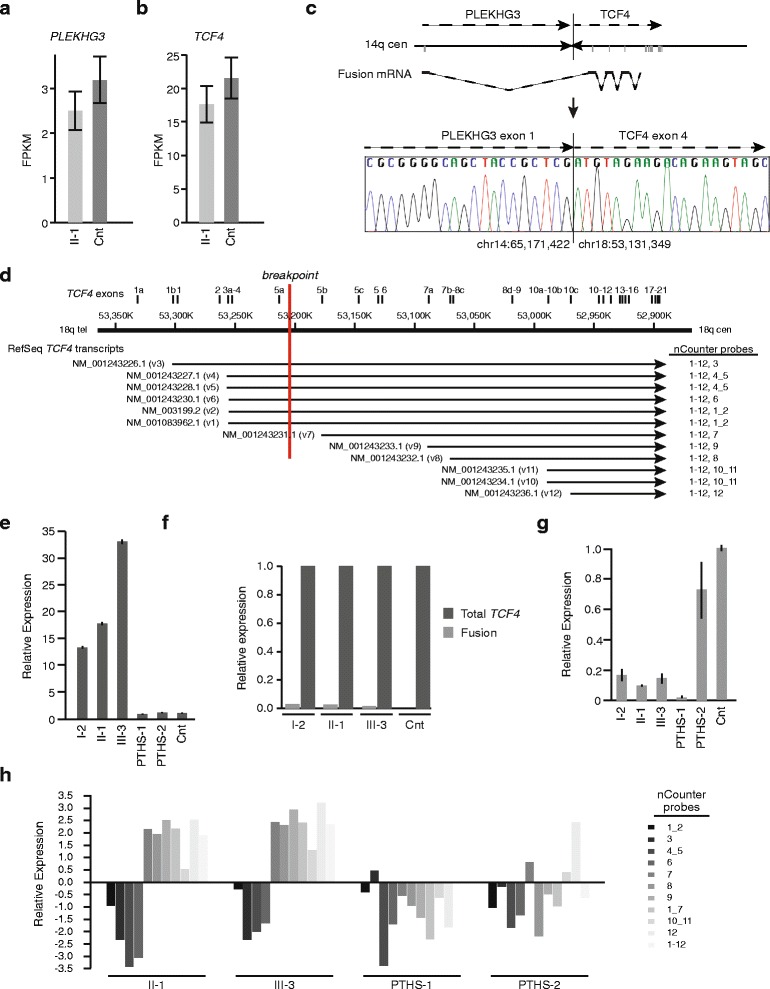
Analysis of expression of genes at the breakpoints, i.e., *PLEKHG3* and *TCF4*. (**a**) Graph comparing *PLEKHG3* expression between cultured skin fibroblasts of individual II-1 and cultured skin fibroblasts of an unaffected control. Analysis was done by quantitation of transcriptome sequence reads and is shown as fragments per kilobase of exon per million fragments mapped (FPKM). (**b**) Graph comparing combined levels of all *TCF4* transcript variants between cultured skin fibroblasts of individual II-1 and cultured skin fibroblasts of an unaffected control. Analysis was done by quantitation of transcriptome sequence reads. (**c**) Figure showing the *PLEKHG3 : TCF4* fusion transcript generated by the derivative chromosome 14. The first non-coding exon of *PLEKHG3* (3’ end chr14:65,171,422) is spliced to a coding exon of *TCF4* (5’ end chr18:53,131,349). This coding exon is incorporated into *TCF4* transcripts NM_001243227.1, NM_001243226.2, NM_001243228.1, NM_001083962.1, NM_001243230.1, and NM_003199.2. Transcriptome sequencing detected the fusion transcript in cultured skin fibroblasts (data not shown) and RT-PCR and Sanger sequencing detected it in peripheral blood. (**d**) Diagram showing the 12 *TCF4* RefSeq transcripts aligned to chromosome 18 as annotated in GRCh37/hg19. Physical positions and *TCF4* exons along chromosome 18 are shown at the top; the exons are labeled as per Sepp et al. [15]. The breakpoint within *TCF4* is shown in red. The transcript variant number is shown in parentheses following each RefSeq accession number. nCounter probes detecting each transcript are shown in the right-hand column. (**e**) Graph showing the composite mRNA level of all 12 TCF4 transcripts in the peripheral blood among individuals II-1, I-2, III-3, two individuals with Pitt-Hopkins Syndrome (PTHS) and pooled unaffected controls. Measurement was done by qRT-PCR. (**f**) Graph comparing the level of *PLEKHG3 : TCF4* fusion mRNA to the composite mRNA level of all 12 TCF4 transcripts in the peripheral blood of individuals II-1, I-2, III-3, and pooled unaffected controls. Measurement was done by qRT-PCR. (**g**) Graph comparing the mRNA level in peripheral blood for transcripts interrupted by the translocation (NM_001243226.1, NM_001243227.1, NM_001243228.1, NM_001243230.1, NM_003199.2, NM_001083962.1) inclusive of the *PLEKHG3-TCF4* fusion transcript. The mRNA levels were measured among individuals II-1, I-2, III-3, two individuals with Pitt-Hopkins Syndrome (PTHS) and pooled unaffected controls by qRT-PCR. (**h**) Graph comparing mRNA levels in peripheral blood for various TCF4 transcripts as assessed by nCounter Analysis. The mRNA levels were measured for individuals II-1 and III-3, two individuals with Pitt-Hopkins Syndrome (PTHS) and pooled unaffected controls. The transcripts detected by each nCounter probe are defined in panel **d**

Given the lack of regional gene expression changes on chromosomes 14 and 18, we focused on gene expression patterns at the chromosomal rearrangement breakpoints to look for evidence of proximal-regulatory effects [38]. We tested 14 genes around each balanced translocation breakpoint (Table 2). Of the genes tested on chr14, *PLEKHG3* was unaltered and *HSPA2*, 164 kb upstream of *PLEKHG3*, was marginally down-regulated at 53.2 fragments per kilobase of exon per million fragments mapped (FPKM) vs. 120.3 FPKM for the control (*P-*value < 5×10^−05^). Of the genes tested on chr18, *DNYAP* was marginally up-regulated at 2.6 FPKM vs. 0 FPKM (*P-*value < 5×10^−05^) and *TCF4, CCDC68,* and *RAB27B* had reduced expression. Total *TCF4* expression was 70-80 % of the unaffected control. This level of total *TCF4* mRNA was confirmed by qRT-PCR (Fig. 4a-b).

**Table 2:** Expression of genes near the chromosome 14 and 18 breakpoints

*Chromosome 18*							
Gene	Chromosome	UDP4637 FPKM	Control FPKM	log (UDP/Control)	*p*-value	q-value	Significant
MBD2	chr18	10.0	9.0	−0.15	0.250	0.39	no
SNORA37	chr18	0.0	0.0	0.00	1.000	1.00	no
POLI	chr18	3.7	2.7	−0.47	0.001	0.00	yes ^a^
STARD6	chr18	0.0	0.0	0.00	1.000	1.00	no
C18orf54	chr18	3.0	1.4	−1.09	0.000	0.00	yes ^a^
DYNAP	chr18	2.6	0.0	N/A	0.000	0.00	yes ^a^
RAB27B	chr18	0.6	1.7	1.52	0.000	0.00	yes ^a^
CCDC68	chr18	0.3	1.1	2.02	0.000	0.00	yes ^a^
TCF4	chr18	17.6	21.5	0.29	0.026	0.06	no
LOC100505474	chr18	0.0	0.0	0.00	1.000	1.00	no
TXNL1	chr18	22.5	25.7	0.19	0.155	0.27	no
WDR7	chr18	6.7	6.4	−0.08	0.563	0.70	no
BOD1P	chr18	0.0	0.0	inf	1.000	1.00	no
*Chromosome 14*							
Gene	Chromosome	UDP4637 FPKM	Control FPKM	log (UDP/Control)	*p*-value	q-value	Significant
TEX21P	chr14	0.1	0.1	−0.04	1.00	1.00	no
MTHFD1	chr14	32.0	29.4	−0.12	0.36	0.51	no
AKAP5	chr14	0.1	0.5	2.15	0.00	0.00	yes ^a^
ZBTB25	chr14	2.4	2.5	0.08	0.66	0.78	no
ZBTB1	chr14	13.2	14.6	0.15	0.28	0.42	no
HSPA2	chr14	53.3	120.4	1.18	0.00	0.00	yes
PPP1R36	chr14	0.3	0.3	0.16	0.71	0.81	no
PLEKHG3	chr14	2.5	3.2	0.35	0.02	0.05	yes ^a^
SPTB	chr14	0.0	0.1	0.74	1.00	1.00	no
CHURC1,CHURC1-FNTB,FNTB	chr14	20.3	20.4	0.01	0.97	0.98	no
GPX2	chr14	0.0	0.0	inf	1.00	1.00	no
LOC100506321	chr14	0.0	0.1	1.83	1.00	1.00	no
MAX	chr14	23.2	18.8	−0.31	0.26	0.40	no
MIR4706	chr14	0.0	0.0	0.00	1.00	1.00	no
RAB15	chr14	3.4	2.3	−0.54	0.23	0.37	no

^a^ FPKM value too low to interpret as significant (as evaluated by cufflinks)

### Other genomic variants do not explain the phenotype

These expression results suggested that a mutation other than chromosomal translocation might be responsible for the observed phenotype. To identify potential pathogenic single-nucleotide variants (SNVs), small insertions, deletions, and genomic copy number aberrations, we integrated data from the short-insert library whole-genome sequencing and SNP chip analysis. Concordance of array- and sequence-based SNP calling exceeded 99.2%. Bases within genes and their corresponding exons exceeded 98 % coverage with each base sequenced >10 fold on average. We identified a total of 3.6 million single nucleotide differences (>Q20; heterozygote/homozygote ratio = 1.6; transition/transversion ratio = 2.05) between the proband genome and the human reference sequence (NCBI build 37; hg19). Most SNVs (>94 %) were common variants in the general population, and 1.6 % of the SNVs localized to exonic regions. Of the exonic SNVs, 461 of these were unreported or had a frequency of <0.1 % in dbSNP. We ranked these 461 variants by various pathogenicity prediction software including CDPred [41] and PolyPhen2 [42]. None of these candidate variants showed potential to cause ID (data not shown). In the absence of another likely strong candidate variant to explain the phenotype of the patients, we concluded that the disruptions of *TCF4* or *PLEKHG3* remained the most likely causes.

Analysis of the sequence data from the disrupted genes found that *PLEKHG3, DYNAP,* and *TCF4* had no missense changes. *TCF4* had two heterozygous synonymous polymorphisms, rs1261084 and rs1261085*.*

### Altered expression of TCF4 is the most likely cause of milder form of ID

A recent study reported a patient with a chromosomal translocation disrupting *TCF4* and a phenotype milder than PTHS [43]. Because this report attributed the mild phenotype to expression of a *TCF4* fusion transcript, we analyzed cultured skin fibroblasts for expression from the *TCF4* locus. The derivative chromosome fusing *PLEKHG3* intron 1 (NM_001308147.1; chr14: 65,191,597) to *TCF4* intron 3 (NM_001083962.1; chr18: 53,200,017) is compatible with generation of a fusion transcript initiating at the *PLEKHG3* transcriptional start site and extending from exon 4 through the remaining exons of *TCF4* (NM_001083962.1, TCF4-B+); this fusion transcript has potential to encode a protein initiating in exon 4 of *TCF4* (Additional file 1: Figure S4). To test for such a fusion transcript, we mapped the mate-pairs from the RNASeq data, described above, against human reference sequence (NCBI 37, hg19) with TopHat. Gene expression was evaluated with Cufflinks and mate-pairs were categorized as (a) mapping to the same gene or (b) mapping to different genes on different chromosomes. This detected a gene-fusion between exon 1 of *PLEKHG3* (chr14: 65,171,193-65,171,422) and exon 4 of *TCF4* (NM_001083962.1; chr18: 53,131,307-53,131,368); 4 paired-end reads and 11 single reads spanned the junction (TopHat fusion and BLAT alignment of unaligned reads) (data not shown). This analysis did not detect a fusion transcript between *TCF4* and *DYNAP*. RT-PCR of skin fibroblast total RNA and Sanger sequencing of the products confirmed the *PLEKHG3-TCF4* fusion transcript (Fig. 4c) and the absence of a *TCF4*-*DYNAP* fusion transcript (data not shown). Contrary to the hypothesis that the *PLEKHG3-TCF4* fusion transcript contributed substantial *TCF4* transcripts, the RNASeq analysis detected few fusion mRNAs.

To determine if the paucity of fusion transcripts was an artifact of cell culture, we tested cDNA derived from peripheral blood by qRT-PCR. Quantitation of the 12 RefSeq TCF4 transcripts (Fig. 4d) showed that total *TCF4* mRNA levels in the blood of the patients were 14–33 fold higher than for unaffected controls (Fig. 4e) and that the fusion transcripts from the derivative chromosome constituted only 2-3 % of the total *TCF4* expression for all transcripts (Fig. 4f). Focusing on transcripts interrupted by the translocation (NM_001243226.1, NM_001243227.1, NM_001243228.1, NM_001243230.1, NM_003199.2, NM_001083962.1) (Fig. 4d), qRT-PCR of cDNA derived from blood showed that mRNA levels for these transcript variants, inclusive of the *PLEKHG3-TCF4* fusion transcript, were expressed at only 10-20 % of the level of the control (Fig. 4g). We concluded therefore that expression of a fusion transcript did not rescue overall *TCF4* expression [43].

Because *TCF4* has promoters distal to the translocation breakpoint, we hypothesize that the rescue of *TCF4* expression and that the moderation of the patient phenotype arises from increased expression of these shorter transcripts. To test this, we compared RNA extracted from blood of II-1 (UDP_4637) and III-3 (UDP_4765), PTHS controls and unaffected controls using an nCounter Gene expression assay with probes distinguishing many *TCF4* transcripts (Additional file 1: Table S6). Compared to the unaffected controls, the patient blood RNA had increased levels of total *TCF4* mRNA and of transcripts (NM_001243231.1, NM_001243233.1, NM_001243232.1, NM_001243235.1, NM_001243234.1, NM_001243236.1) initiating downstream of the translocation breakpoint, whereas it had decreased or unchanged levels of transcripts (NM_001243226.1, NM_001243227.1, NM_001243228.1, NM_001243230.1, NM_003199.2, NM_001083962.1) initiating upstream of the translocation (Fig. 4h). Compared to the unaffected controls and as predicted for nonsense mediated mRNA decay, the two individuals with PTHS had decreased levels of mRNA for most *TCF4* transcripts (Fig. 4e, h).

To determine whether the upregulated transcripts arose from the translocated chromosome, we performed digital droplet PCR for expression of rs1261085, a SNP within the 3′ UTR of all *TCF4* transcripts and for which the propositus’ father is heterozygous. Using cDNA derived from blood of II-1, we found that half of the *TCF4* mRNA was derived from the derivative chromosome and half from the wildtype allele (data not shown).

## Discussion

We demonstrate that a chromosomal translocation interrupting proximal *TCF4* segregates with mild ID and defines a genomic interval critical for this phenotype versus PTHS. Additionally, we find that although such translocations can produce fusion transcripts, increased transcription from *TCF4* promoters distal to the breakpoint likely ameliorates the phenotype, i.e. prevents the congenital anomalies and neurologic co-morbidity typical of PTHS.

Despite the disruption of *TCF4*, the individuals reported herein did not meet the diagnostic criteria for PTHS (Table 1) [32, 44]. Using two PTHS clinical scoring systems, the affected individuals considered herein had a clinical score of only 1 on the system of Marangi et al., in which a minimum score of 10 is an indication for *TCF4* mutation analysis [44], and they had 0 out of 20 criteria on the system of Whalen et al. in which a score of >15 is an indication for *TCF4* mutation screening [32].

To understand better the genotype-phenotype correlation, we analyzed the transcripts affected by translocations causing PTHS versus mild ID [43, 45, 46]. Using the *TCF4* structure defined by Sepp et al*.* [30], the translocation of our patient and the patient reported by Schluth-Bolard et al*.* suggest that disruption of all transcripts originating *at and proximal* to the exon 8 promoters is sufficient to cause PTHS [46], but only disruption of transcripts originating proximal to the exon 8 promoters associates with mild ID. In other words, the minimal set of intact *TCF4* transcripts for mild ID are NM_001243231.1, NM_001243233.1, NM_001243232.1, NM_001243235.1, NM_001243234.1, and NM_001243236.1. In contrast, if transcripts NM_001243231.1, NM_001243233.1 and NM_001243232.1 are disrupted along with transcripts NM_001243226.1, NM_001243227.1, NM_001243228.1, NM_001243230.1, NM_003199.2 and NM_001083962.1, the phenotype is PTHS [44, 46]. Affirming this genotype-phenotype correlation, PTHS-associated missense, nonsense, splice site, frame-shift, and deletion mutations minimally alter the transcripts disrupted by the PTHS-associated translocations [30].

Transcripts originating at and proximal to the exon 8 promoters contain a nuclear localization signal (NLS), transcription activation domain (AD) 2 and the basic helix-loop-helix domain, whereas transcripts initiating at the exon 10 promoters do not contain an NLS. Transcripts containing the NLS encode products predominantly localized to the nucleus, whereas those products without an NLS are distributed between the nucleus and cytoplasm [15]. Consequently, we hypothesize that partial phenotypic rescue from PTHS to mild ID occurs by increased expression of TCF4 isoforms localizing to the nucleus. Supporting this, point mutations associated with PTHS generally occur within or downstream of the NLS, whereas point mutations associated with mild ID generally occur upstream of the NLS [30, 47, 48]. An exception to this generalization is the mutation NM_001083962:c.[C469T];[=] (p.R157*) that alters the first amino acid of the NLS and can cause either mild ID or PTHS [47, 49]. We must acknowledge that expression profile of *TCF4* in brain may differ from that in other tissues and that a potential shortcoming of our study, as well that of many others, is reliance on expression analysis of blood and skin fibroblasts.

Besides delineating a minimal set of mutated transcripts for occurrence of PTHS, the translocations in our patients and the individual reported by Kalscheuer et al*.* [43] show that biallelic expression for all *TCF4* transcripts is essential for full intellectual function. The diminution of longer TCF4 isoforms is not rescued by increased expression of the shorter isoforms. This raises at least three possible disease mechanisms for consideration: 1) AD1, which is encoded only in the longer transcripts, is essential for full neural function of TCF4; 2) the longer transcripts have promoters preferentially active in neural tissues; and 3) the overexpression of shorter isoforms induces mild ID. Supporting the first, AD1 and AD2 act synergistically for transcriptional activation compared to AD1 or AD2 alone [15]. Minimizing the likelihood of the second, although not excluding it, transcripts initiating at the exon 10 promoters, not those initiating proximal to exon 8, are those most highly expressed in studied brain regions [15]. Supporting the third, a gain-of-function disease mechanism is consistent with prior studies of PTHS-associated *TCF4* mutations [30]. We conclude therefore that both gain- and loss-of-function mechanisms might contribute to *TCF4*-associated mild ID caused by chromosomal translocation and that expression of the full complement of *TCF4* transcripts at the appropriate dosage is required for full intellectual function.

Related to the role of TCF4 for maintenance of intellectual function and variability of expressivity within a family, the three generations described herein provide some insight. All three affected individuals had similar intellectual disability suggesting minimal variation in expressivity. Also, the absence of early cognitive decline in the adult individuals suggests that TCF4 dysfunction is most detrimental during early brain development.

## Conclusions

In summary, this study of a *TCF4* translocation and its consequence on *TCF4* promoter usage and fusion transcript expression provides insight into the relative roles of TCF4 isoforms in ID, highlights the potential for some TCF4 isoforms to partially rescue the dysmorphisms and ID characteristic of PTHS, shows that the ID phenotype associated with *TCF4* mutation can be relatively consistent over generations and from childhood through adulthood. Validation of these observations in other patients is, however, required.

### Availability of supporting data

The transcriptome data set supporting the results of this article are available in GEO repository, series record GSE77742 (http://www.ncbi.nlm.nih.gov/geo/query/acccgi?acc=GSE77742).

### Ethics, consent and permissions

The family described herein gave consent to study participation.

## Additional files

Additional file 1: Supplementary Methods, Tables and Figures. (PDF 2983 kb) Additional file 2: Read summary statistics of whole-genome sequencing on Illumina HiSeq 2000 (>Q20). (XLSX 56 kb) Additional file 3: CASAVA human reference sequence (hg19) coverage summary of whole genome library. (XLSX 51 kb)

## Abbreviations

AD1: transcription activation domain 1
AD2: transcription activation domain 2
bHLH: basic helix-loop-helix
bp: base pairs
CCDC68: coiled-coil domain containing 68
CDPred: Conserved Domain-based Prediction
CNS: central nervous system
DYNAP: dynactin associated protein
FPKM: fragments per kilobase of exon per million fragments mapped
HASH1: achaete-scute family bHLH transcription factor 1
ID: intellectual disability
Kb: kilobases
Math1: atonal bHLH transcription factor 1
Mb: megabases
neuroD2: neuronal differentiation 2
NLS: nuclear localization signal
Olig2: oligodendrocyte lineage transcription factor 2
PLEKHG3: pleckstrin homology domain containing, family G
PolyPhen2: Polymorphism Phenotyping v2
PTHS: Pitt-Hopkins syndrome
RAB27B: RAB27B, member RAS oncogene family
RefSeq: NCBI Reference Sequence Database
RNASeq: RNA sequencing
SNV: single nucleotide variant
TCF4: transcription factor 4
