# Supplementary material for: Complex translocation disrupting TCF4 and altering TCF4 isoform expression segregates as mild autosomal dominant intellectual disability
Source: Orphanet J Rare Dis. 2016 May 14;11:62. doi: 10.1186/s13023-016-0439-6 (PMC4868023; doi:10.1186/s13023-016-0439-6)
Supplement: Additional file 1: — Supplementary Methods, Tables and Figures. (PDF 2983 kb) [file 13023_2016_439_MOESM1_ESM.pdf]

Complex Balanced Translocation Disrupting *TCF4* and Altering TCF4 Isoform Expression Segregates as Mild Autosomal Dominant Intellectual Disability

Valerie Maduro[1], Barbara Pusey[1], Praveen F. Cherukuri[1], Paul Atkins[2,3], Christèle du Souich[2,3], Rosemarie Rupps[2,3], Marjolaine Limbos[4], David R. Adams[1], Samarth S. Bhatt[5], David Chai[6], Patrice Eydoux[6], Anna Lehman[2,3], Amanda E. Links[1], May C. Malicdan[1], Christopher E. Mason[7], Marie Morimoto[2,3], James C. Mullikin[8], Andrew Sear[9], Clara Van Karnebeek[3], Pawel Stankiewicz[5], William A. Gahl[1, 10], Camilo Toro[1], Cornelius F. Boerkoel[1,2,3]

[1]NIH Undiagnosed Diseases Program, Common Fund, Office of the Director, NIH, National Institutes of Health, Bethesda, Maryland, USA

[2]Department of Medical Genetics, [3]Child and Family Research Institute, University of British Columbia, Vancouver, Canada

[4]Sunny Hill Health Centre for Children, Vancouver, Canada.

[5]Department of Molecular and Human Genetics, Baylor College of Medicine, Houston, USA

[6]Department of Pathology and Laboratory Medicine, University of British Columbia, Vancouver, British Columbia, Canada.

[7]Institute for Computational Biomedicine, Weill Cornell Medical College, Department of Physiology and Biophysics, New York, NY 10021

[8]NIH Intramural Sequencing Center, National Human Genome Research Institute, NIH, Bethesda, Maryland, USA

[9]Department of General Practice, Faculty of Medicine, University of British Columbia, Vancouver, British Columbia, Canada

[10]NHGRI, National Institutes of Health, Bethesda, MD USA

Abstract: 219 words

Text: 5,608

Corresponding author:

Cornelius F. Boerkoel, M.D., Ph.D.
University of British Columbia
Department of Medical Genetics
Children's and Women's Health Centre of BC
4500 Oak St., Rm., C234
Vancouver, B.C. V6H 3N1
Canada
Phone: 604-875-2157
Fax: 604-875-2376
Email: cboerkoel@gmail.com

SUPPLEMENTARY METHODS

Analysis of gene expression on chromosomes 14 and 18

The Pearson correlation coefficient of gene expression on chromosomes 14 and 18 was calculated using all-possible pairs (N^2) resulting from a window of 3 genes to derive matrix M. M is symmetric; therefore, correlation values $M_{i,j}=M_{j,i}$. Values in M range from -1 to +1, where -1 occurs when gene expression values are anti-correlated and +1 when their expression patterns are correlated.

The comparison is between two conditions A, and B; A is the normal control fibroblast gene expression data, and B is individual II-1 fibroblast gene expression data. Values for gene expression are FPKM values derived from RNASeq data. A window was defined as adjacent genes along the same chromosome. All genes in a window were treated as a vector, and the correlation coefficient was calculated as follows, e.g., window of 3. Window range = {1,NumberOfTotalGenes (chromosome)}:

Window of three genes in two different conditions

$A=a_1, a_2, a_3$

$B=b_1, b_2, b_3$

Vector average calculation:

$$\overline{a_i} = \frac{1}{w}\sum_{k=0}^{w-1} a_{i+k} \quad \text{[e.g: } \overline{a} = (a_1 + a_2 + a_3)/3\text{]}$$

$$\overline{b_i} = \frac{1}{w}\sum_{k=0}^{w-1} b_{i+k} \quad \text{[e.g: } \overline{b} = (b_1 + b_2 + b_3)/3\text{]}$$

Deviation from the mean

$$\overline{A} = (a_1 - \overline{a}, a_2 - \overline{a}, a_3 - \overline{a})$$

$$\overline{B} = (b_1 - \overline{b}, b_2 - \overline{b}, b_3 - \overline{b})$$

Calculate Pearson correlation coefficient (PCC) for each gene at position p

$$PCC = \frac{\overline{A}.\overline{B}}{EucD_A * EucD_B}$$

Where,

$$\overline{A}.\overline{B} = \sum_{k=0}^{w-1} (a_k - \overline{a})(b_k - \overline{b})$$

$$EucD_A = \sqrt{\sum_{k=0}^{w-1} (a_k - \overline{a})^2}$$

$$EucD_B = \sqrt{\sum_{k=0}^{w-1} (b - \overline{b})^2}$$

SUPPLEMENTARY TABLES

Supplementary Table 1. Read summary statistics of whole-genome sequencing on Illumina HiSeq 2000 (>Q20). See attached excel file.

Supplementary Table 2. CASAVA human reference sequence (hg19) coverage summary of whole genome library. See attached excel file.

Supplementary Table 3. Summary statistics of homozygous and heterozygous single nucleotide variants (>Q20) detected in whole-genome sequencing.

Chromosome	Hom	Het	HetNonRef	Total(Hom,Het)	Het-Hom-Ratio
chr1	114,372	170,990	122	285,362	1.49503375
chr2	116,706	180,975	94	297,681	1.550691481
chr3	95,962	154,014	81	249,976	1.604947792
chr4	109,890	158,219	138	268,109	1.43979434
chr5	81,931	143,658	72	225,589	1.753402253
chr6	84,378	152,063	107	236,441	1.802164071
chr7	78,386	127,937	70	206,323	1.632140944
chrX	73,785	7,234	27	81,019	0.098041607
chr8	74,595	118,256	91	192,851	1.585307326
chr9	59,963	96,305	83	156,268	1.606073745
chr10	71,624	109,301	61	180,925	1.526038758
chr11	73,860	112,252	80	186,112	1.519794205
chr12	66,278	103,879	55	170,157	1.56732249
chr13	61,146	81,180	41	142,326	1.327642037
**chr14**	**48,230**	**68,465**	**40**	**116,695**	**1.419552146**
chr15	44,528	64,492	43	109,020	1.448347107
chr16	42,095	73,528	45	115,623	1.746715762
chr17	36,542	65,022	28	101,564	1.779377155
**chr18**	**42,863**	**63,732**	**50**	**106,595**	**1.486876794**
chr20	26,467	51,907	22	78,374	1.961196962
chrY	1,084	4,646	8	5,730	4.28597786
chr19	28,162	50,215	29	78,377	1.783076486
chr22	16,578	31,750	19	48,328	1.915188804
chr21	20,557	37,784	28	58,341	1.838011383
**All Chrom**	**1,469,982**	**2,227,804**	**1,434**	**3,697,786**	**1.515531483**
**Autosomes**	**1,395,113**	**2,215,924**	**1,399**	**3,611,037**	**1.588347324**
**chrM**	**36**	**1**	**0**	**37**	**0.027777778**

Supplementary Table 4. Sequences of oligos used for amplifying breakpoints from genomic DNA

Primer Name	Sequence
junct-1a 18-14F	GAAGGCTGGTACCAAGGAAAGC
junct-1a 18-14R	CCAAGAAGTTTATAGTCCAATCCTGGTG
junct-2a 18-14 F	AAATAGATTGGGCTCAGTGGGATTC
junct-2a 18-14 R	CCAACACACACACCATTAGGGAGAT
IntraChromFusion_1F	ACCATGTGAAAGTCAGATTTTTATT
IntraChromFusion_1R	CCCATGTCATACATGTCAAGATT

Supplementary Table 5. Sequences of Oligos used for RT-PCR and qRT-PCR

Primer Name	Sequence	RT-PCR	qRT-PCR
TCF4v2RevCompF	GCAAGTGGACATTTTACTGGC		X
TCF4v2LongFormA2R	GCTGGTCATGTGGTCATAGG		X
NM_001243234ShortFormF	GGCGGCAACTCTTTGATGTA		X
NM_001243234ShortFormR	TAGGGAAAGTGCTGGTTGCT		X
IntraChromFusion_1F	ACCATGTGAAAGTCAGATTTTTATT	X	
IntraChromFusion_1R	CCCATGTCATACATGTCAAGATT	X	
Fusion1aPLEKHG3ex1TCF4ex4F	CCCCTTCAGAGAGCGACTTT	X	
Fusion1aPLEKHG3ex1TCF4ex4R	CATAGGGAGTCCCATCTCCA	X	

Supplementary Table 6. Nanostring probes

Gene	Accession	Transcript Target Region	Target Sequence	Transcript variants detected	Chromosome 18 (hg19)		RefSeq Transcript ID
					Start	Stop	
TCF4	NM_001083962.1	3136-3235	TTAGGGGAAGCTCGGCT GCCCTAGTAACAAAACC AGCAAACGTCCTGATGA CAACGAAGTGATGACAT TAGCCATTCCTTAGGGTA GGAGGAACAGATGG	1-12	52894659	52894758	NM_001243236, NM_001243235, NM_001243234, NM_001243232, NM_001243233, NM_001243231, NM_001083962, NM_003199, NM_001243230, NM_001243228, NM_001243227, NM_001243226
TCF4	NM_001083962.1	854-953	CCAGCAGGGACCTTGGG TCACATGACAATCTCTCT CCACCTTTTGTCAATTCC AGAATACAAAGTAAAAC AGAAAGGGGCTCATACT CATCTTATGGGAG	1-7	53070713	53128312	NM_001243226, NM_001243231, NM_001243230, NM_001243228, NM_001243227, NM_003199, NM_001083962
TCF4	NM_001243226.1	188-287	CTCAGGACATTGTAACAT GCACTTGGGTTGAGAACT GCTACTCGAGCTTCTCCA GGAGGCCCTTGGAGCAA ATGTTTTGTAAACACCAA TCTAAGAACAT	3	53298588	53303001	NM_001243226
TCF4	NM_001243227.1	13-112	AGCCCGCAGTTCCCGGAT GTGAATGGATTACAATGT ATCTTTCAGGGAAACCTA TTATTATCAATGTGACTC CACGGGGGAGTCCATGG TGATGATGATG	4 & 5	53256934	53257033	NM_001243228, NM_001243227
TCF4	NM_001243230.1	111-210	CTGAACTGTTCAGGCTTC AGATTGTAACTGACGATC TGAGGAAAAATGAGATG [illegible]	6	53252534	53255750	NM_001243230

TCF4	NM_001243231.1	150-249	AGTGGGAAAAATGGACC AACTTCTTTGGC GGAATTTATTACTTCCTA TTCATATGGAAGAGGAC AGCAGAGATGTAGAAGA CAGAAGTAGCTCAGGGT CCTGGGGGAATGGAGGA CATCCAAGCCCGTC	7	53131311	53177851	NM_001243231
TCF4	NM_001243232.1	214-313	TTAGAGAAAGTGGAGGC CATTGGAATGACAGTTTT TGGAAGTGTGGAGCAGT TTGGCTAAGAATAGGAA TGAAGGATATTTTTTTCC AGTTTATCATAGC	8	53070914	53071013	NM_001243232.1
TCF4	NM_001243233.1	223-314	CCACATGCTTTTTGGCGA CTCTTCATCACGTATTTG GGCTTAGATCTTCTGGTG CCTCCCTCGGCGTGCTAC CCATAAGGTCGAAAGAA AAGTAAAACAG	9	53089410	53089501	NM_001243233
TCF4	NM_001243235.1	103-192	GAAAAAAGAATCCGAGA CCATGTACTGCGCATACA CAATCCCGGGCATGGGC GGCAACTCTTTGATGTAC TACTATAATGGGAAAGC GGTCTATGCTCCA	10 & 11	52988899	52988988	NM_001243235, NM_001243234
TCF4	NM_001243236.1	105-204	CATGTCTGACCAATCAGC AAACAACACCACAGGAT GAAATTTAAACAATGCA GATGCTCAGATACAGGG CTATGTTGCCTGGACCAC GAAGGGAAAGCAG	12	52969649	52969748	NM_001243236
TCF4	NM_003199.2	111-210	CTGAACTGTTCAGGCTTC AGATTGTAACTGACGATC TGAGGAAAAATGAGGTG CTCGATGAATTTTCGTTT GTATTTTTTGGCGAGGCG	1 & 2	53255651	53255750	NM_003199, NM_001083962

GAPDH	NM_002046.3	973-1072	GGGGAGGTGTT CACTCCTCCACCTTTGAC GCTGGGGCTGGCATTGCC CTCAACGACCACTTTGTC AAGCTCATTTCCTGGTAT GACAACGAATTTGGCTAC AGCAACAGGG

SUPPLEMENTARY FIGURES

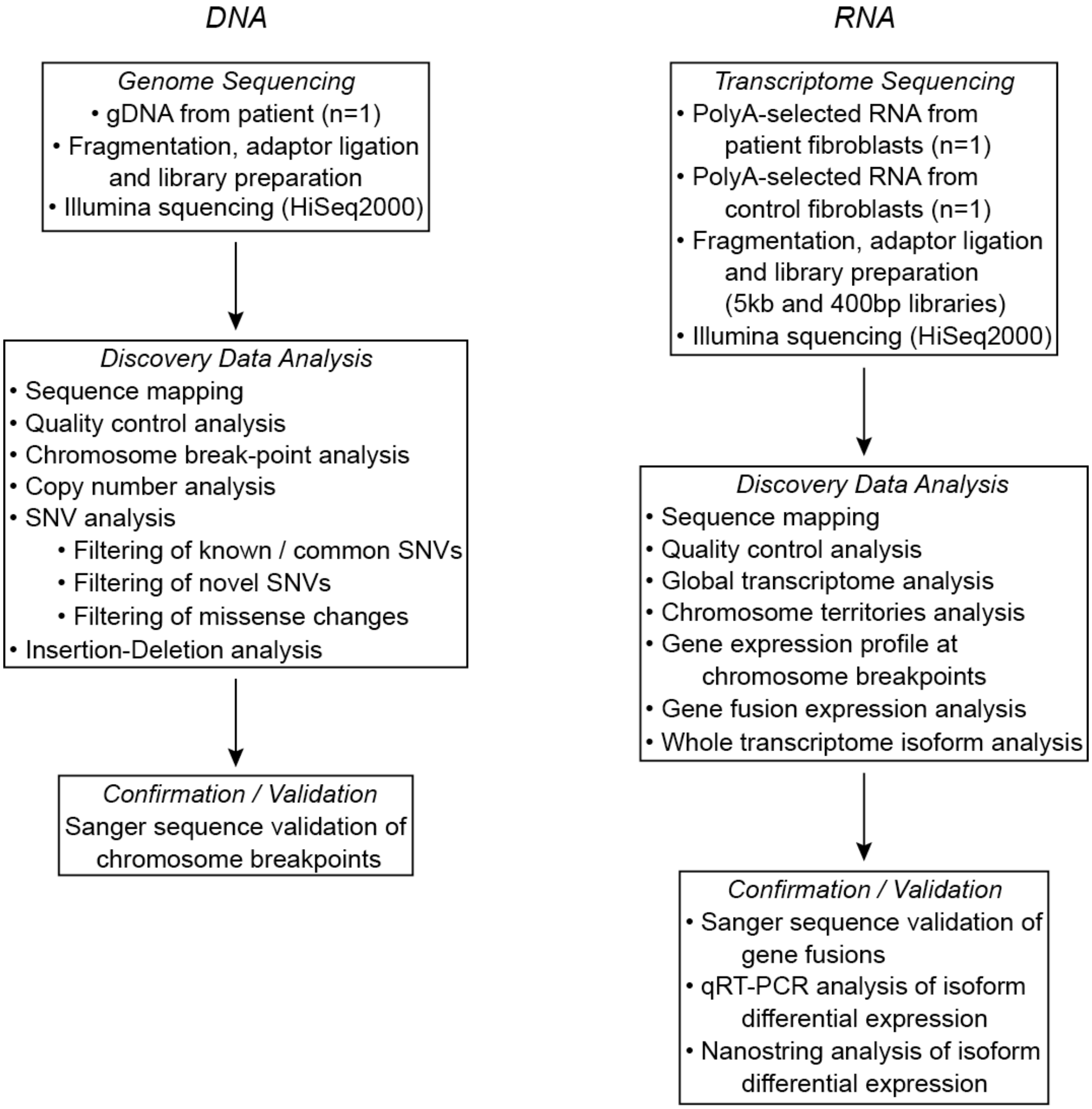

Supplementary Figure 1. Experimental design of next-generation sequencing analysis of the whole-genome and whole-transcriptome of patient (and corresponding control data). Schematic overview of comprehensive analysis of patient genome and transcriptome data to identify and discover disease-causing genes, and unravel their relative gene expression patterns. Genomic DNA was extracted from blood, and total RNA for polyA-selected RNASeq analysis was extracted from skin fibroblast cells of patient and control normal cells.

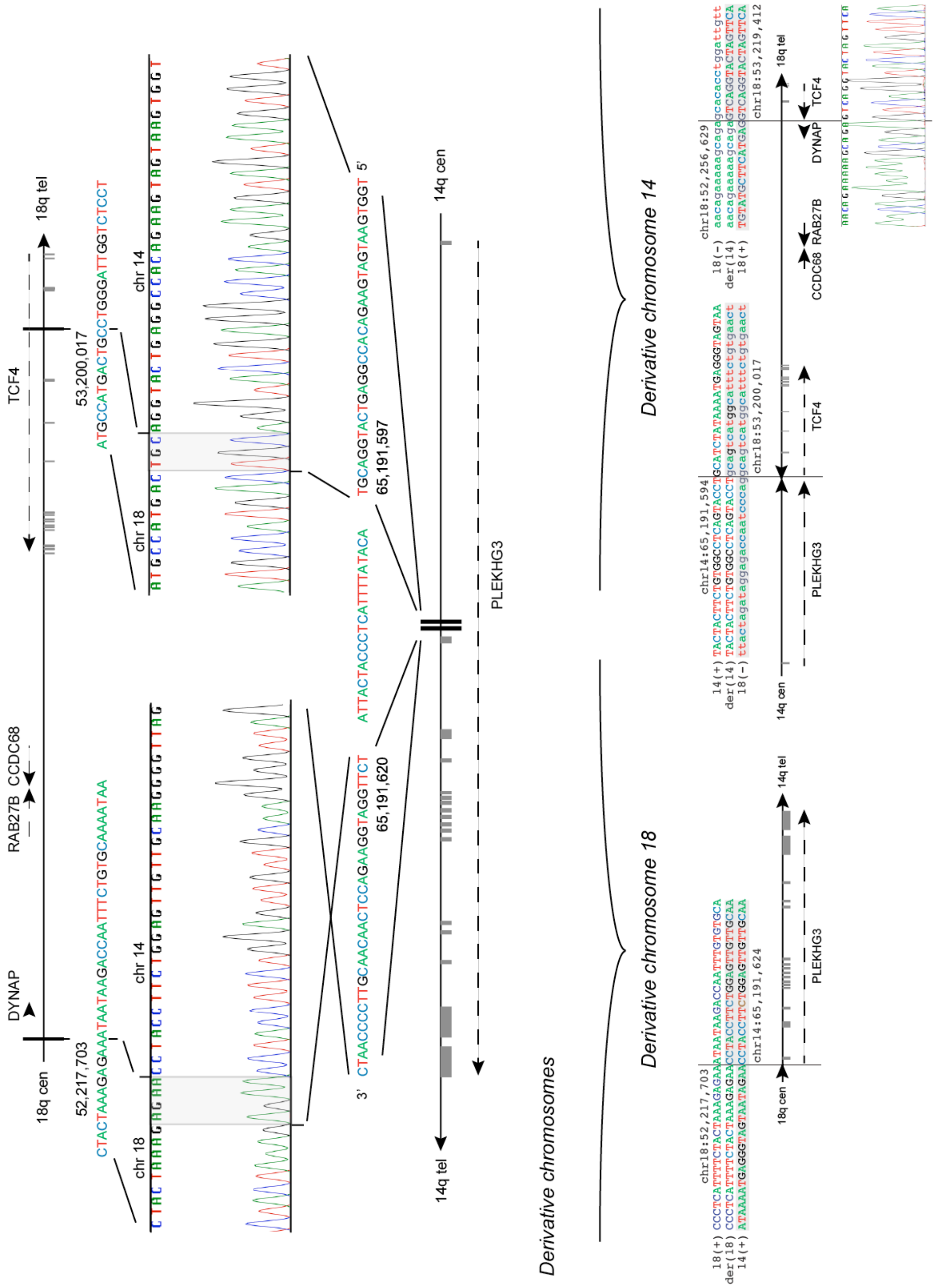

Supplementary Figure 2. Diagram of the chromosome 14 and 18 breakpoints and chromatograms showing the sequence across the breakpoint junctions. Note that chromosome 18 has two breakpoints and an inversion to give rise to the two derivative chromosomes. All numbering of physical positions is in hg19. Note that each junction is associated with a deletion as described in the text. The joining of the double strand chromosome 14 and 18 breaks likely occurred by microhomology; the regions of

microhomology are highlighted in gray.

A

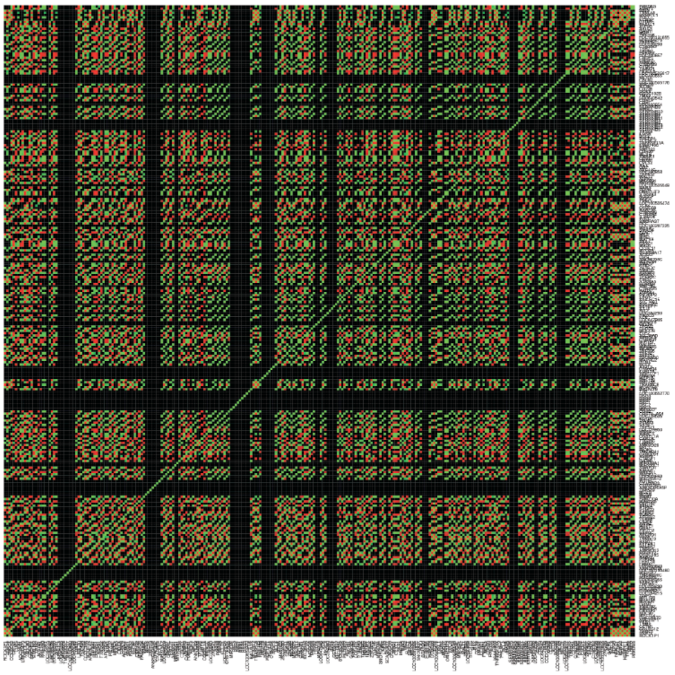

B

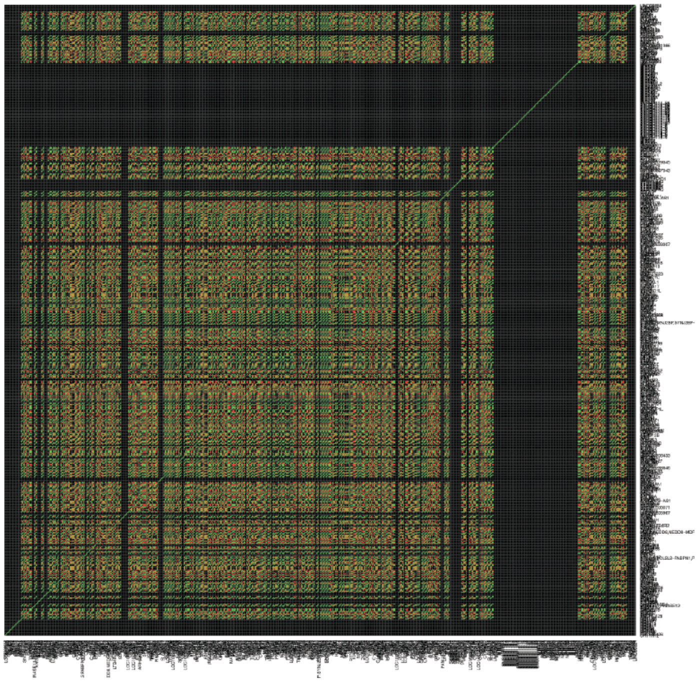

Supplementary Figure 3. Pearson correlation-coefficient matrices for all pairs of genes on chromosome 18 (A) and chromosome 14 (B). Using $M_{i,j} = M_{j,i} = 1$ for positive correlation and $M_{i,j} = M_{j,i} = -1$ for anti-correlation of gene-expression, the topological overlap along the diagonal did not identify signatures of anti-correlation along the entire length of chromosomes 18 and 14. This suggests that alterations in gene expression are not a result of changes in chromatin territory for each derivative chromosome.

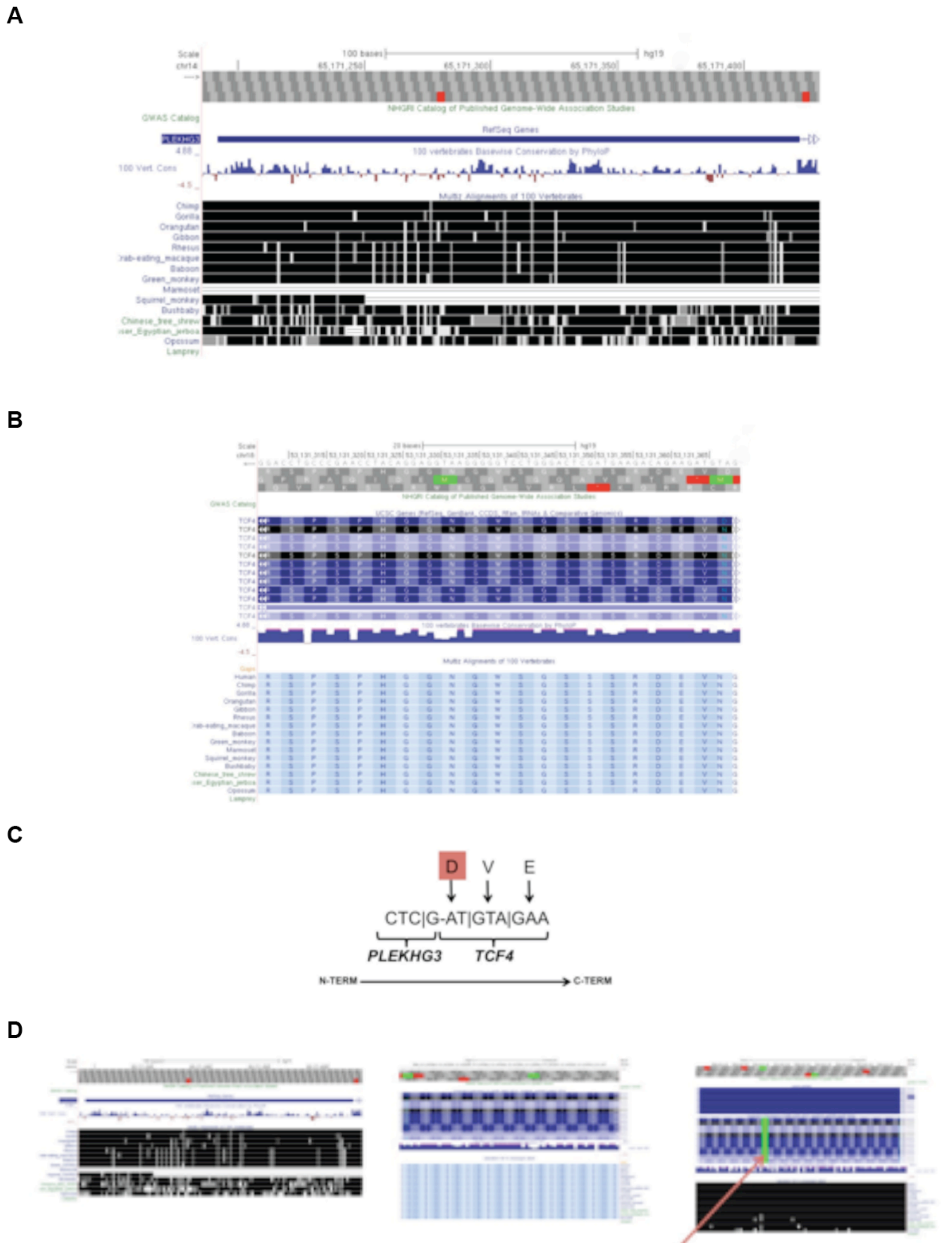

Supplementary Figure 4. Evaluation of exons of *PLEKHG3* (exon 1; UTR) and *TCF4* (exon4; coding) potential for coding a gene fusion product. (A) Exon 1 of *PLEKHG3* which is a 5' untranslated region (UTR); shown here are all three protein coding frames, and non of these three show a methionine (*green*). (B) Exon 4 of *TCF4* which codes for

protein sequence (*top frame*); however it does not have a Methionine. (C) This panel shows a hypothetical scenario where an Aspartate (*D*) would be substituted at the junction (instead of a highly conserved Asparagine (*N*) in case exon4 is translated in the fusion product. There is evidence for the amino acid Methionine in exon5 of TCF4 that could lead to a potential short-isoform of TCF4. (D) This is a hypothetical depiction of the fusion gene product originating in exon5 of TCF4 giving rise to a shorter version of TCF4 instead of the original longer forms of TCF4.
